# Misplacement of a Port Catheter: A Differentiated View

**DOI:** 10.1155/2017/1640431

**Published:** 2017-12-26

**Authors:** Christoph Evers, Angelos Gazis, Wendy Thuss-Patiance, Albrecht Kretzschmar

**Affiliations:** ^1^Department of Radiation Oncology, University of Halle, Halle (Saale), Germany; ^2^Department of Medical Oncology, St. Georg Hospital Leipzig, Leipzig, Germany; ^3^Department of Radiology, St. Georg Hospital Leipzig, Leipzig, Germany; ^4^Department of Pneumology, Protestant Lung Hospital Berlin, Berlin, Germany

## Abstract

Clinical radiological controls after the insertion of central venous catheters (CVC) are of high importance. Misplacement of the CVC, outside of large vessels, as described in our first case, occurs in more than 7% of cases and may be associated with life-threatening events. A persistent left-sided superior vena cava (PLSSVC) occurs in 0.3–0.5% of the standard population. In one of the cases a CT scan of the chest showed the catheter in a PLSSVC. Neoadjuvant radiochemotherapy was indicated in a patient with an adenocarcinoma of the oesophagus. Under hospitalised monitoring, full-dose chemotherapy was given. Consequences for the patients arise when the findings are known for future interventions. If a PLSSVC is expected and a CVC is to be inserted, the venous return to the heart should be evaluated first, to preclude a possible backflow to the left atrium. With this constellation, a right-to-left shunt can be expected in in 10% of cases. Affected patients face a high risk of developing cardioembolic events.

## 1. Overview

Clinical radiological controls after the insertion of central venous catheters (CVC) are of high importance. The CVC should be positioned in the superior vena cava above the right ventricle. The thrombogenicity there is reduced because of the fast flow [1]. Misplacement of the CVC, outside of large vessels, as described in our first case, occurs in more than 7% of procedures and may be associated with life-threatening events [2]. Congenital abnormalities, as mentioned in our second case, are possible as well. Imaging demonstrated a persistent left-sided superior vena cava (PLSSVC). PLSSVC occurs in 0.3–0.5% of the standard population and in 4–12% of individuals with a congenital heart malformation. In 75% of all cases, PLSSVC is an incidental finding [3]. Patients likely to have a PLSSVC are difficult to identify on the basis of a medical history alone.

## 2. Case Report 1

**Figure 1:**
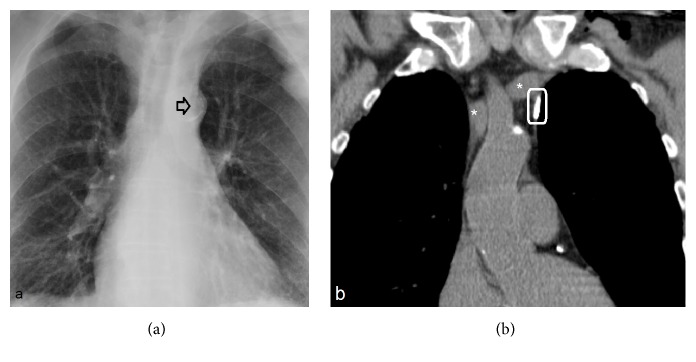
(a) The a.p. chest X-ray shows the catheter tip (black arrow) at the left margin of the mediastinum. (b) depicts a CT of the thorax. The catheter tip is marked in white. ^*∗*^left brachiocephalic vein.

A central venous catheter was implanted in a 77-year-old patient (56 kg, 168 cm, BMI: 20 kg/m^2^) suffering from metastatic rectal carcinoma. After insertion, the catheter became infected. 14 days after removal, a new port catheter system (PCS) (powerPort ChronoFlex, Bard, Salt Lake City, USA) was inserted. Under ultrasound-guidance, the catheter was directed into the left brachiocephalic vein. Normal saline solution was injected without any complications. The a.p. chest X-ray showed an atypical position of the catheter tip at the left margin of the mediastinum (Figure 1(a)).

Computer tomography (CT) of the thorax revealed misplacement of the port catheter. It was placed outside the large vessels (Figure 1(b)). Distal to the catheter tip, a vessel, with the size of approximately 1 mm, was seen. The catheter was removed and a new catheter was placed using the Seldinger technique.

## 3. Case Report 2

**Figure 2:**
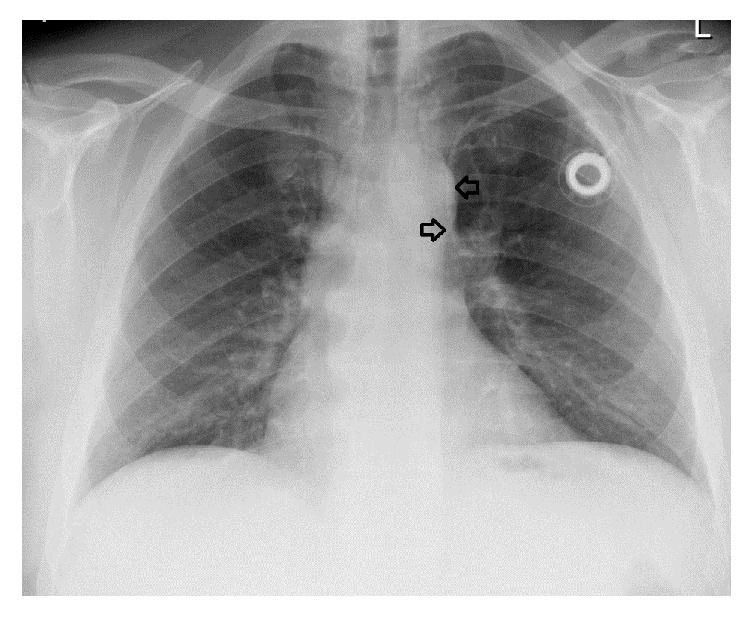
The a.p. chest X-ray shows the port catheter in the left-sided superior vena cava (black arrows).

**Figure 3:**
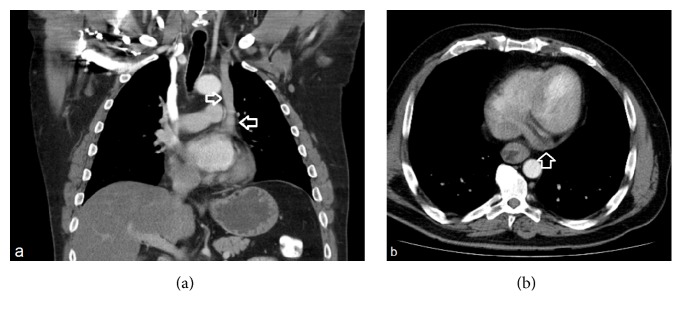
CT of the thorax, including coronal and axial cuts. In picture (a), white arrows show the passage of the PLSSVC while in picture (b) the ending in the coronary sinus is depicted.

Neoadjuvant radiochemotherapy was indicated in a 50-year-old patient (105 kg, 190 cm, BMI: 29 kg/m^2^) with a moderately differentiated adenocarcinoma of the lower third of the oesophagus. As the patient was an enthusiastic table tennis player and right-handed, the PCS (powerPort ChronoFlex, Bard, Salt Lake City, USA) was inserted on the left side. The postoperative examination showed an atypical position of the catheter tip at the left margin of the mediastinum (Figure 2). However, blood could be aspirated. A reevaluation of a past CT scan of the chest showed the catheter in a PLSSVC (Figures 3(a) and 3(b)).

The flushing of the catheter did not lead to any adverse events. An echocardiogram with intravenous contrast medium confirmed the PLSSVC with its typical ending in the coronary sinus. A right-to-left shunt was not demonstrated. Under hospitalised monitoring, full-dose chemotherapy with fluorouracil as a 24-h continuous infusion, folic acid, oxaliplatin, and docetaxel were given. There were neither subjective complaints nor rhythm disturbances. The patient received four cycles of neoadjuvant and four cycles of postoperative chemotherapy via the port without any complication.

## 4. Discussion

An understanding of embryological development is essential for understanding the events described here. Between the 7th and 8th week of gestation, a crosslink between the right and the left anterior cardinal vein arises. Increased blood flow to the right cardinal vein occurs and allows its increase in caliber. Those parts, which are caudal to the crosslink, obliterate. If not, a PLSSVC results [3]. This vein extends perpendicularly anterior to the aortic arch, as well as to the pulmonary artery and lateral to the vagus nerve. In the case of a PLSSVC, with its typical ending in the coronary sinus, rhythm disturbances and mechanical complications may occur after catheterisation. In 75% of all cases a PLSSVC is an incidental finding. This might be due to the fact that, in 80–92% of all patients, the vein drains without haemodynamic relevance into the coronary sinus [3].

At times, however, the vein drains into the left atrium which is the only real malposition resulting in a right-to left shunt. In our case, there were no signs that would have led to such conclusions. In the remaining 92% of cases, the PLSVC drains into the coronary sinus. A CVC placed in the PLSVC can, thus, be used without risk, ultrasound clinical judgement assumed. In Table 1, all types of superior vena cava are presented.

Consequences for the patients arise when the findings are known for future interventions. However, it is not just the risk of arrhythmias which, indeed, is increased, that should be held in mind, but also that thermodilution measurements can be altered. Here, an abnormal thermodilution curve can be seen. Accordingly derived haemodynamic data could be irregular and should then not be used for treatment.

An atypical position of the catheter end at the left margin of the mediastinum may be caused, apart from misplacement in a PLSSVC, by a misplacement in small veins. These include the left superior intercostal vein, the left internal thoracic vein, and the left pericardiacophrenic vein [4].

With a perforation of small veins, life-threatening complications such as pericardial tamponade, hydrothorax, or hydromediastinum may occur [5]. Vein stenosis and thrombi in smaller vessels, caused by applying hyperosmolar solutions, are further complications [4].

## 5. Conclusion

A differentiated consideration of port catheter misplacement in supposedly identical cases is obligatory. Routine checks contribute to the patient's protection. Informing a patient explicitly about his aberrant vein anatomy may prevent life-threatening complications.

## Figures and Tables

**Table 1:** Upper vena cava morphology (SVC) [6].

Type I	Normal anatomy with only one right-sided SVC

Type II	Abnormal anatomy with only one left-sided SVC. There is no right-sided SVC and patients present with a CXR with a noticeably small mediastinal shadow (right SVC absent)

Type III	Abnormal anatomy with a right- and a left-sided SVC
Type IIIa	The left brachiocephalic vein, connecting left and right SVC, is present
Type IIIb	The brachiocephalic vein is missing and consequently the connection between the two SVC
